# Quantitative Proteomic Approach Targeted to Fibrinogen β Chain in Tissue Gastric Carcinoma

**DOI:** 10.3390/ijms19030759

**Published:** 2018-03-07

**Authors:** Ombretta Repetto, Stefania Maiero, Raffaella Magris, Gianmaria Miolo, Maria Rita Cozzi, Agostino Steffan, Vincenzo Canzonieri, Renato Cannizzaro, Valli De Re

**Affiliations:** 1 Facility of Bio-Proteomics, Immunopathology and Cancer Biomarkers, CRO Aviano National Cancer Institute, 33081 Aviano, Italy; orepetto@cro.it; 2 Gastroenterology, CRO Aviano National Cancer Institute, 33081 Aviano, Italy; smaiero@cro.it (S.M.); raffaella.magris@cro.it (R.M.); rcannizzaro@cro.it (R.C.); 3 Medical Oncology, CRO Aviano National Cancer Institute, 33081 Aviano, Italy; gmiolo@cro.it; 4 Immunopathology and Cancer Biomarkers, CRO Aviano National Cancer Institute, 33081 Aviano, Italy; mrcozzi@cro.it (M.R.C.); asteffan@cro.it (A.S.); 5 Pathology, CRO Aviano National Cancer Institute, 33081 Aviano, Italy; vcanzonieri@cro.it

**Keywords:** DIGE, comparative proteomics, gastric cancer, fibrinogen β chain, FGB, coagulation, platelets, biomarker

## Abstract

Elevated plasma fibrinogen levels and tumor progression in patients with gastric cancer (GC) have been largely reported. However, distinct fibrinogen chains and domains have different effects on coagulation, inflammation, and angiogenesis. The aim of this study was to characterize fibrinogen β chain (FGB) in GC tissues. Retrospectively we analyzed the data of matched pairs of normal (N) and malignant tissues (T) of 28 consecutive patients with GC at diagnosis by combining one- and two-dimensional electrophoresis (1DE and 2DE) with immunoblotting and mass spectrometry together with two-dimensional difference in gel electrophoresis (2D-DIGE). 1DE showed bands of the intact FGB at 50 kDa and the cleaved forms containing the fragment D at ~37–40 kDa, which corresponded to 19 spots in 2DE. In particular, spot 402 at ~50 kDa and spots 526 and 548 at ~37 kDa were of interest by showing an increased expression in tumor tissues. A higher content of spot 402 was associated with stomach antrum, while spots 526 and 548 amounts correlated with corpus and high platelet count (>208 × 10^9^/L). The quantification of FGB and cleaved products may help to further characterize the interconnections between GC and platelet/coagulation pathways.

## 1. Introduction

Gastric cancer (GC) is the fifth most common cancer and the third leading cause of cancer death in the world [1]. Therefore, there is a great interest in deciphering the molecular pathways associated with its progression and prognosis. Environment and lifestyles are general risk factors for GC, but interaction of diet with multiple genetic and epigenetic alterations also occur during GC development [2,3,4,5]. Proteomics provided consistent information in revealing proteome alterations associated with GC, dissected some of the mechanisms underlying gastric cancerogenesis, and enabled the identification of several diagnostic, prognostic, and predictive biomarkers [6,7,8,9].

Hemostasis systemic perturbations are well known to occur in GC [10]. In particular, venous thromboembolism (VTE) has been implicated in GC progression and metastasis [11,12,13,14]. Clinically relevant VTE in GC patients shows an incidence of >5% at 1 year post diagnosis and 12–17% at 2 years, increasing to 24.4% in metastatic advanced GC [15,16,17].

Most recently, by using a two-dimensional difference in gel electrophoresis (2D-DIGE)-based comparative proteomic approach on human tissues we identified the fragment D of fibrinogen ß chain (FGB) as marker of preoperative response to neoadjuvant chemo-radiotherapy in rectal cancer [18]. FGB protein is linked to α and γ chains by numerous disulfide bonds to form fibrinogen, a key molecular player of both coagulation and inflammation [10,19,20]. The mean pre-treatment plasma fibrinogen level has been correlated with a hyper-coagulable status, tumor progression and prognosis of several types of malignancies (ovarian [21], biliary [22], esophageal [23], pancreatic [24]), including GC [10,11,12,13,14].

At present, the complex molecular interplay between fibrinogen and cancer has not been fully analyzed locally within the tumor tissue. 

In the present study, we investigated differential FGB protein expression levels in biopsies of non-metastatic GC patients, to assess interconnections between GC and platelet/coagulation pathways, and to propose FGB as molecular marker for GC diagnosis. Correlations among the means of platelet (PLT) count, white blood cell count (WBC), neutrophil-to-lymphocyte ratio (NLR) and FGB cleaved products were also analyzed.

## 2. Results

### 2.1. Patients and Disease Characteristics

A total of 28 consecutive patients with GC were included in our analysis. Characteristics of both patients and the disease are reported in Table 1 and Table A1. Patients’ tumors were mostly characterized by corpus location (65%), diffuse histotype (65%), and T3–T4 pathological stages (68%).

The means of plasma routine blood count parameters at diagnosis are reported in Table 2 (detailed information in Table A2).

### 2.2. One-Dimension Immunoblotting

**Figure A1:**
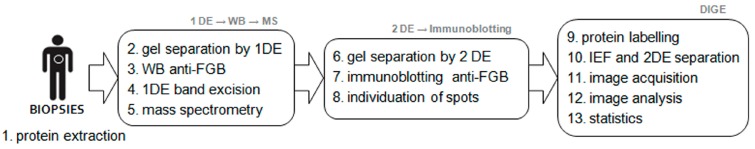
A schematic illustration of the proteomic workflow adopted. Proteins have been extracted from both gastric tumor-affected (T) and normal (N) biopsies of 28 patients. After extraction (1), some protein pools were separated by one-dimensional electrophoresis (1DE) (2), the presence of fibrinogen β chain (FGB) was investigated by western blotting (WB) (3), and the presence of FGB in the cross-reacting 1DE bands was validated by mass spectrometry (4,5). Some protein extracts resulted to contain high levels of FGB were separated by two-dimensional electrophoresis (2DE) (6), and the presence of FGB was successfully individuated to some spots after 2DE immunoblotting on gels (7, 8). For comparative analyses by DIGE, all the protein extracts were labelled (9), separated by isoelectric focusing (IEF) and molecular weight (2DE) (10), images were acquired (11) and targeted image analyses were performed on the only spots of interest containing FGB (12,13). Details are explained in Methods section.

The quantitative proteomics workflow adopted is illustrated in Figure A1. 

**Figure 1:**
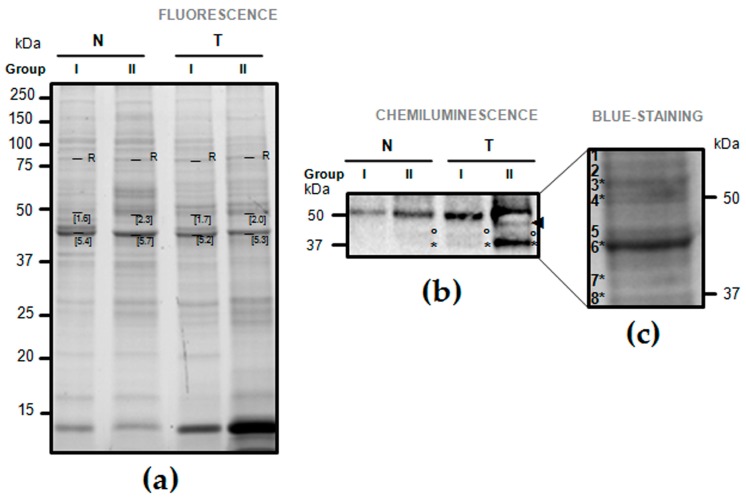
One-dimensional electrophoresis (1DE) and immunoblotting of fibrinogen ß chain (FGB) in normal (N) versus tumor-affected (T) gastric tissues belonging to patients divided according to their tumor stage (Group I: T1–T2; Group II: T3–T4). (**a**) Image of the 1DE stain-free gel fluorescence acquired upon excitation with the Chemidoc system before its transfer to nitrocellulose membrane. Numbers refer to the relative quantity of the band calculated with the Image Lab^TM^ software (R, reference band for which quantity is 1); (**b**) Chemiluminescence signals of proteins cross-reacting with the anti-FGB antibody. Circles and asterisks evidence a band at ~40 and 37 kDa, respectively, while the arrow shows a band at <50 kDa; (**c**) From the blue-stained 1DE gel in (**a**), an area of ~37–60 kDa was excised, and gel portions numbered 1 to 8 submitted to analysis by mass spectrometry for protein identification. Asterisks confirmed the presence of FGB product(s) in the gel portions.

**Figure A2:**
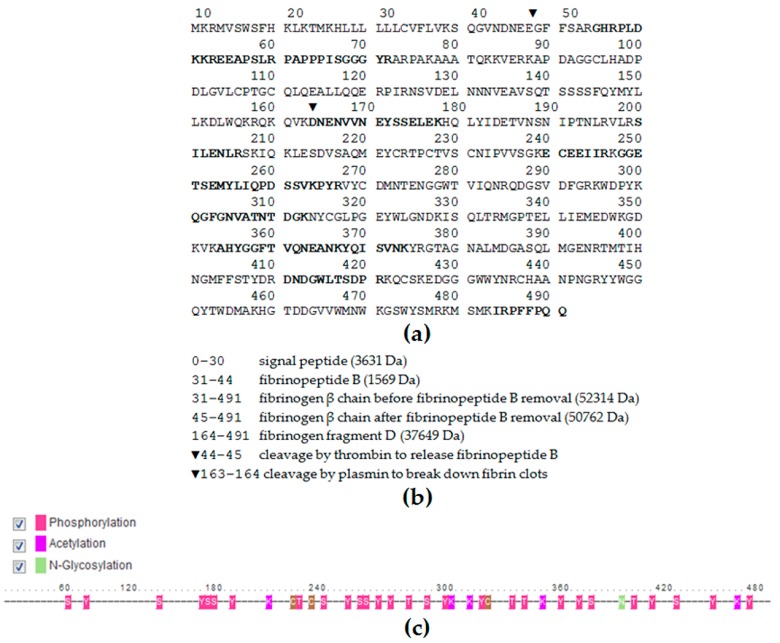
The complete amino acid sequence of fibrinogen β chain (FGB) immature form (http://www.uniprot.org/uniprot/P02675), where the peptides identified by mass spectrometry in gel portions of Figure 1c are evidenced in bold (a). The FGB immature form of 56 kDa is processed into a mature form of ~52 kDa after signal peptide removal. During the conversion of fibrinogen into fibrin, thrombin cleaves fibrinopeptide B from β chains and releases FGB forms of ~51 kDa, which can be further cleaved by plasmin into fibrinogen fragments D of ~37 kDa to break down fibrin clots. These processes are summarized in (b). All the possible post-translational modifications (phosphorylation, acetylation and *N*-glycosilation) are shown as interactive sequence in (c), after interrogation of P02675 as substrate with iPTMnet bioinformatic resource (http://proteininformationresource.org/iPTMnet).

In T versus N tissues of patients belonging to both groups of tumor staging (Group I: T1 and T2; Group II: T3 and T4), a higher content of a band at ~50 kDa, corresponding to the weight of the entire FGB (UniProtKB entry: P02675; Figure A2), occurred (Figure 1b). Two additional bands of ~37 and ~40 kDa were detected in T tissues, with a higher content in Group II patients especially for the 37 kDa band (Figure 1b). One-dimensional electrophoresis revealed that the amount of total protein loading among samples was homogeneous (Figure 1a).

### 2.3. Validation of Fibrinogen β Chain (FGB) by Mass Spectrometry

The presence of FGB in the 1DE portions of the ~37–60 kDa area (Figure 1c) was validated by mass spectrometry. FGB peptides were identified at ~50 kDa (Figure 1c, gel portions nr 3 and 4), ~40 kDa (Figure 1c, gel portions nr 6), and ~37 kDa (Figure 1c, gel portions nr 7 and 8) (Table A3). The FGB identified at ~50 kDa was the entire form after release of fibrinopeptide B by thrombin in 44–45, and the identified peptides covered from 45 to 491 (Table A3, Figure A2). The FGB identified at ~37 kDa corresponded to the fragment D, after plasmin cleavage of FGB in 163–164, and the identified peptides covered from 164 to 491 (Table A3). Fragment D of FGB was also identified in the band at ~40 kDa, and its slightly different molecular weight (MW) may be related to possible post-translational modifications such as glycosylation, as evidenced after interrogation with iPTMnet bioinformatic resource (Figure A2).

### 2.4. Cross-Reaction of FGB on Two-Dimensional Electrophoresis (2DE) Maps of Gastric Tissue

**Figure 2:**
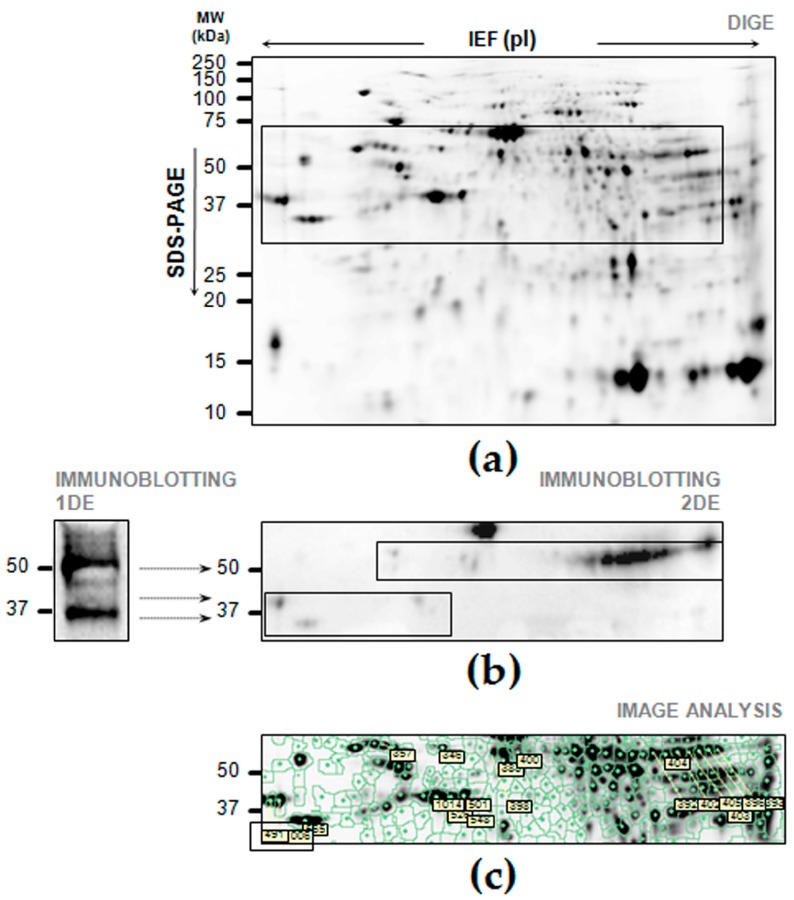
Two-Dimensional Electrophoresis (2DE) and immunoblotting of FGB from proteins pooled from normal and tumor-affected gastric tissues. (**a**) After labelling with cyanines, proteins were resolved by isoelectrofocusing (IEF) over the pI 3–10, followed by 8–16% gradient Sodium Dodecyl Sulfate—PolyAcrylamide Gel Electrophoresis (SDS-PAGE). The gel image was acquired and added to a Decyder project before its transfer to a nitrocellulose membrane. The frame corresponds to the gel area shown in (**b**); (**b**) Visualization of spots cross-reacting with the anti-FGB antibody ([1F9], GeneTex) in the gel area corresponding to the rectangle in (**a**). The 2DE protein map showed FGB cross-reacting spots in the same molecular weights (MWs) as those evidenced by immunoblotting on 1DE gel, here visualized on the left (for more details, see Figure 1b). The frames corresponds to the gel area containing the FGB cross-reacting spots shown in (**c**), on which our image analysis focussed; (**c**) FGB cross-reactive spots were identified, numbered, and analyzed using the Decyder software for quantitative analysis and comparison, as described in Methods section.

An immunoblotting performed to detect FGB on 2DE proteome map of gastric tissues is illustrated in Figure 2. The matching between the spots cross-reacting with anti-FGB antibody on the nitrocellulose membrane and the protein profiles of the 2DE gel, from which the membrane was obtained, was digitally performed. A total of 19 matched spots were individuated, and their differential patterns analyzed in a 2D-DIGE project comparing all the 25 gels (Figure 2c; Table A1). 

Among the 19 spots cross-reacting with the FGB antibody, a part of them was present in the MW region of ~50 kDa and another one in the MW region of ~37–40 kDa (Figure 2b). 

In particular, a total of 10 cross-reacting spots (numbered as spots 392, 393, 396, 397, 398, 100, 402, 403, 404 and 405) were found in the MW of 50 kDa as multiple pI isoforms, coming from different phosphorylation status, as evidenced after interrogation with iPTMnet bioinformatic resource (Figure A2). The interrogation of P02675 sequence (UniProtKB) with PhosphoSite Plus evidenced a total of 43 possible different pI values depending on 43 different phoshorylated residues (results in: https://www.phosphosite.org/isoelectricCalcAction.action?id=20774&residues=43&x=41&y=3; accessed on 18 December 2017). The MW/pI position of spots/isoforms corresponding to the entire FGB form in our 2D map fitted with others’ published findings as well as with the calculated MW/pI 52.314/7.15. While the position of the FGB cleaved spots/isoforms fitted with our previous findings in rectal tissues [18], as well as with the calculated MW/pI 37.649/5.85.

### 2.5. Differential Expression of FGB in Gastric Cancer Tissues

Among the 19 spots cross-reacting with the FGB antibody, a total of 13 spots (numbered as spot 383, 393, 396–398, 402–405, 501, 526 and 548) were present in all the gels and, thus, further analyzed. 

The results of variation in their abundance between normal and tumor tissues are reported in Table 3, where significant changes at *p* < 0.05 are evidenced in bold. Six among these spots (393, 402, 404, 405, 526 and 548) were differentially expressed between N and T tissue in both Group I and Group II.

**Figure 3:**
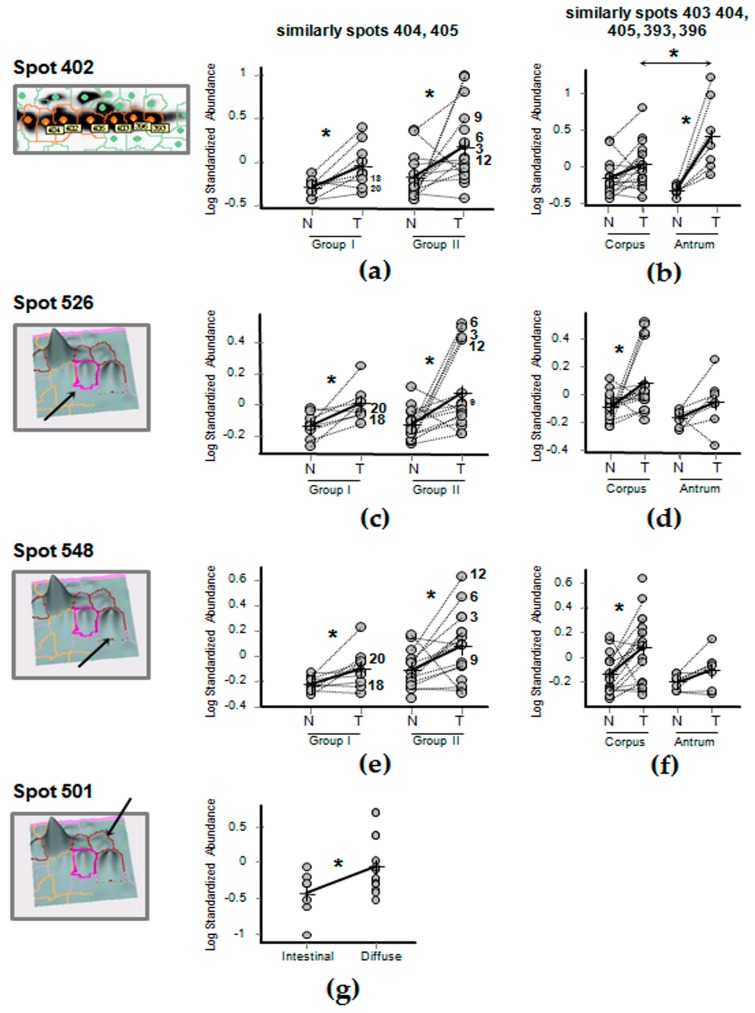
Graphical visualization of abundance distribution of spots 402, 526, 548 and 501 in tumor non-affected (N) and tumor-affected (T) gastric tissue biopsies, according to their tumor stage (Group I: T1–T2; Group II: T3–T4), anatomical site (corpus, antrum) or histological type (intestinal, diffuse). (**a**) Abundance of Spot 402, similarly to spots 404, 405, 526 (**c**) and 548 (**d**), increased in T versus N tissues both in Group I and II, and more even in T-tissue of Group II. (**b**) Spot 402 abundance, similarly to spots 403, 404, 405, 393 and 396 was particularly higher in T than N in the antrum location, while spots 526 and 548, showed the highest abundance associated with corpus location. (**e**) Spot 548, similarly to spots 402 (**a**) and 526 (**c**), increased in content in T versus N tissues both in Group I and II, its abundance being higher in T-tissues of Group II. (**f**) Spot 548 content was significantly higher in T versus N tissues in corpus. (**g**) Spot 501 content was higher in diffuse than intestinal histological type. In each graph, a single circle represents the Log standardized abundance of the spot calculated for a single gel/patient. Asterisks indicate a statistically significant difference at paired *t*-test *p* < 0.05 or between T tissues of different groups (**b**,**g**) at Student’s *t*-test *p* < 0.05. Dotted lines combine N and T samples belonging to the same patient and co-migrated within the same gel, while arrows indicate a detail of the Decyder tridimensional 3D view of the graphically visualized spots.

In particular, three spots at ~50 kDa (nr. 402, 404 and 405) and two spots at ~37 kDa (nr. 526 and 548) significantly increased in content in T versus N tissues, as well as, if not statistically significantly, in T of Group II versus T of Group I (Figure 3a,c,e). These spots were, thus, globally associated with tumor and, particularly, in tumor at advanced stages (i.e., T3–T4).

Regarding tumor anatomical sites, spot 402, similarly to spots 403, 404, 405, 393 and 396, showed a significantly higher content in T of antrum (Figure 3b), while spots 526 and 548 showed a significantly higher content in T of corpus (Figure 3d,f).

Regarding tumor histological classification, only the spot 501 showed an association with the diffuse type (Figure 3g).

There was no significant difference in FGB abundance depending on either age (except for spot 402) or sex gender (except for spot 501), in any of the spots analyzed (Table 4).

### 2.6. Correlation of Blood Count Parameters and FGB Abundance

**Figure 4:**
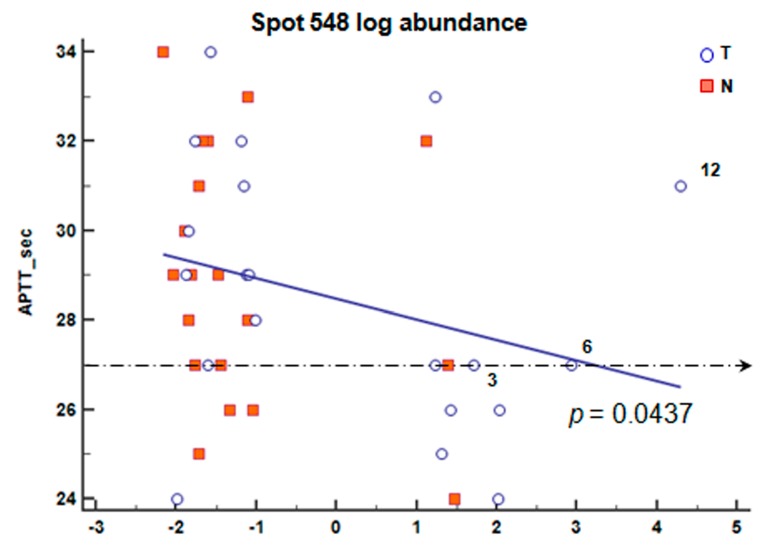
Graphical visualization of the correlation between the activated partial thromboplastin time (APTT) and the log abundance of spot 548 in normal (N, ■) together with malignant (T, ○) tissues of patients belonging to T stage Group II (T3–T4). The broken line indicates the low value considered to be normal. Regression line values were: intercept 28.3 (SE 0.46) and slope −0.55 (SE 0.26).

We first analyzed the differences in blood cell count and coagulation markers with each of the 13 spots of interest. Among these, an inverse correlation between the activated partial thromboplastin time (APTT-sec) and the spot 548 log abundance was found (*p =* 0.05, Figure 4). No significant difference was found between the other blood coagulation markers and FGB spot abundance.

**Figure 5:**
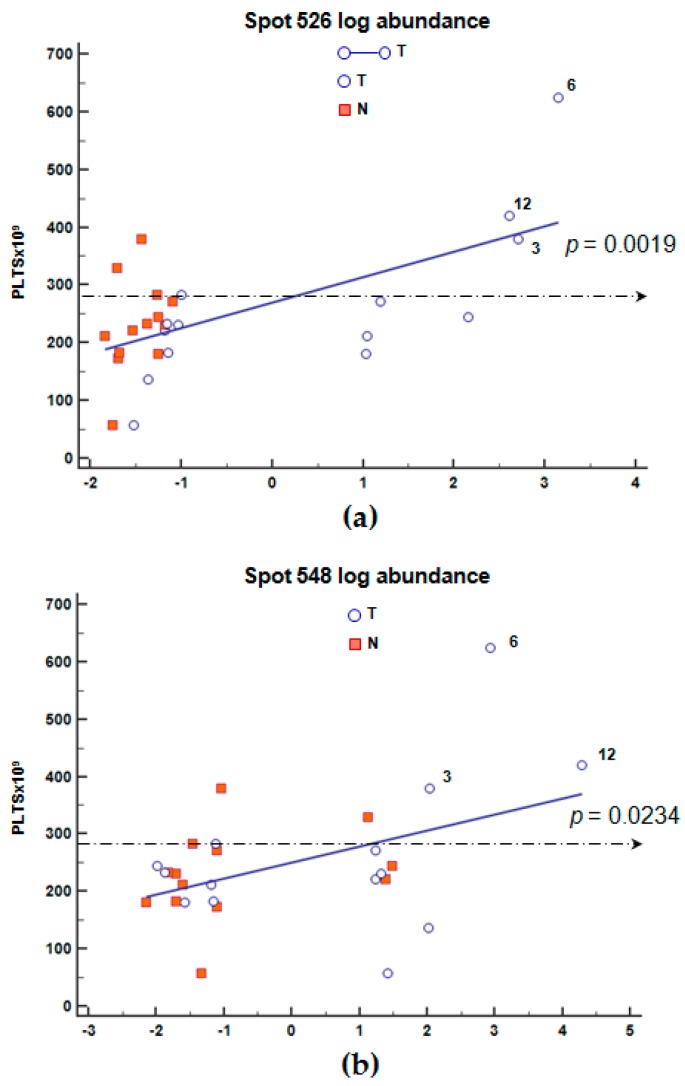
Graphical visualization of the correlation between platelet counts (PLT) and the log abundance of spot 526 in malignant (T, ¡) tissues (**a**), and spots 548 in normal (N, ■) together with malignant (T, ○) tissues of patients (**b**) belonging to Group II (T3–T4). The optimum PLT cut-off of 280 × 10^9^/L(mean ± 70 × 10^9^/L), calculated in the cohort of healthy individuals, is indicated with a broken line. Regression line values were: intercept 260.5 (SE 20.6) and slope 45.0 (SE 12.35) (**a**); intercept 258.4 (SE 15.45) and slope 20.9 (SE 8.85) (**b**).

We further analyzed the association between changes in blood cell count and FGB spot abundance. By focusing on the only tumor stage Group II, there was a significant correlation between PLT count and spot 526 and 548 log abundances (*p* = 0.0006 and *p* = 0.021, Figure 5a,b, respectively).

In a cohort of healthy blood donors (24 females and 22 males), the optimum cut-off value for PLT count was fixed at 280 × 10^9^/L, estimating the sample mean (208 × 10^9^/L) and two standard deviations (±70 × 10^9^/L).

In GC-affected patients, we individuated 5 patients with PLT count >280 × 10^9^/L (patients nr. 3, 6, 9, 12, 18 and 20; Table A2). We excluded patient nr. 11 (PLT count = 330 × 10^9^/L) because it was not possible to separate her low abundant T protein extracts. For both spots, the highest PLT count was found in patients nr. 3, 12 and 6, who showed the highest spot abundance (Figure 5a,b,e).

A positive trend between the other blood parameters and FGB spot abundance was found but without statistical significance.

## 3. Discussion

The present study evidences the differential expression of FGB and its cleaved forms in the biopsies of not-metastatic GC patients. Higher protein contents of the entire form of FGB (~50 kDa) and isoforms containing plasmin-cleavage product “fragment D” of FGB (~37–40 kDa) were found in tumoral gastric tissues compared with non tumoral adjacent tissues, at both early (Group I: T1–T2) and advanced tumor depth (Group II: T3–T4). Even not statistically significant, these increases in content were more evident in patients with advanced GC (Group II: T3–T4) compared with patients at early pathological stages (Group I: T1–T2).

Together with α and β polypeptides, FGB is part of fibrinogen, a disulfide cross-linked homodimer of 340 kDa composed by two outer D domains connected to a central E one. Fibrinogen is known as a principal factor in the maintenance of hemostasis through its conversion from a soluble, plasma protein to an insoluble fibrin gel [18]. During fibrinolysis, both soluble and cross-linked fibrin is enzymatically degraded into fragments, among which the D-dimers typically contains two D domains and one E domain of the original FGB molecule [10]. 

In malignancies, the presence of fibrin(ogen) is known to affect the progression of tumor cell growth and their metastasis. Globally, the assessment of fibrinogen content and fibrinolysis products in plasma is known to help in cancer diagnosis but also to evaluate both therapeutic effects and prognosis. In the particular case of GC, the preoperative plasma content of fibrinogen is clinically relevant as predictor of lymphatic and hematogenous metastasis, tumor progression, as well as tumor stage and survival [10].

Our data evidencing an up-regulation of both FGB and its cleaved fragments in GC tumors versus the adjacent non-tumoral tissues agree with the 2D results from Wang et al. [25] and the label-free quantitative proteomics findings of Dai et al. [26] in six pairs of primary and advanced poorly differentiated gastric adenocarcinoma tissues. On a larger cohort of patients, our study succeeded in confirming this increase in content and strengthening the hypothesis of an association between the accumulation of both FGB and its cleaved products and gastric malignancy directly in tumor tissues.

Our work allowed to evidence a differential accumulation of FGB in gastric tissues of GC patients depending on the anatomical site affected by cancer: the entire FGB was found to be more abundant in the antrum of the stomach, while its cleaved forms showed a higher content in the corpus. This different content of FGB in different stomach regions may reflect different roles played by FGB in tumorigenesis depending on gastric microenvironments and/or their pathogenesis [27]. For instance, *Helicobacter pylori* infection, one of the most important risk factor of GC, is associated with GC of antrum and intestinal type, and it also causes inflammation and gastritis [28], thus it could have an impact on the production of fibrinogen which is strictly related to the inflammatory response(s). 

In the majority of tumor types, abundant fibrinogen, further assembled into fibrin, within the stroma has been proposed to originate from plasma exudation deposition [29]. In solid tumor tissues, the so-called microvascular “enhanced permeability” leads to extravasation and tumoritropic accumulation within the cancer of macromolecules such as plasma proteins, including all those necessary for clot formation (e.g., fibrinogen) [10]. At the same time, an extra-hepatic synthesis of fibrinogen is also known to occur, even if there is still a paucity of information regarding both its structure and function [30,31,32]. The extra-hepatic fibrinogen synthesis may be important for tissue repair at local sites of injury, and/or may have a pathogenetic role, but it is not known whether extra-hepatic synthesis of FGB significantly contributes to the normal plasma fibrinogen concentration. The deposition of fibrin/ogen into the extracellular matrix may form a provisional matrix on which new blood vessels extend, and serve as a scaffold to support binding of tumor growth factors influencing tumor growth, malignant transformation, and migration [33,34,35,36,37].

Interestingly, a study aimed at defining the role of coagulation proteins in tumor progression by immunohistochemistry highly localized fibrin II and fragment D of FGB in the stroma at tumor periphery near the host-tumor interface, and co-localized hemostatic proteins with the “vascular endothelial growth factor” (VEGF), activated by the “tissue factor” (TF) produced by tumor cells [38].

In addition, factors involved in the regulation of fibrin activation/degradation expressed on cancer cell surfaces may also play a role in tumor invasion, proliferation, and metastasis [39,40]. It may thus be tempting in the future to localize FGB and its D fragment in situ in gastric tissues of our cohort of patients, to better understand a possible role played by FGB in cancer progression.

Globally, hyperfibrinogenemia before treatment is increasingly recognized as an important risk factor influencing the survival of patients with solid tumors [41]. In the particular case of GC, hyperfibrinogenemia has been analyzed as prognostic factor of lymphatic and hematogenous metastasis, tumor progression, adjacent organ involvement and survival [42,43,44,45,46,47,48,49,50]. In our cohort of patients, there was a positive trend between fibrinogen level in plasma and FGB spot abundance in the tissues, but this correlation did not reach a statistical significance. Our data are thus insufficient to provide a clear information about a correlation between plasma fibrinogen level and FGB spot content in situ in the tumor microenvironment. 

Nonetheless, we found a negative correlation between spot 548 and APTT, and a positive correlation between spots 526 and 548 and PLT count, with a better correlation between PLTs and spot 526 in the only malignant T tissues. These associations were particularly evident in T tissues of patients with the highest tumor depth (Group II), this suggesting that by increasing tumor depth the content of peptides included in spots 526 and 548 also increased. Normal value of APTT ranged between 27 to 40 s, and measures the time necessary to generate fibrin from initiation of the intrinsic coagulation pathway, thus short APTT is indicative of an increased risk of hypercoagulability. Several studies indicated that hypercoagulability affect tumor cell adhesion and migration across endothelial junctions [51]. Thus, in our series, patients with lower APTT and higher spot 548 values could be considered at higher risk for tumor growth and metastatic dissemination. Of interest, it has been reported that plasma D-dimer, the smallest product resulting from fibrin degradation by enzymes including plasmin, can also interfere with cellular signaling systems, cell proliferation and angiogenesis, but can also affect PLTs and extra-cellular matrix [52,53,54,55]. It has been demonstrated that activation of the coagulation cascade occurring in a cancer such as GC may arise from the direct capacity of tumor cells to express and release pro-coagulant factors, including TF, and to activate the host hemostatic system [56]. 

In our series, patients showing the highest content of the two degraded forms of FGB containing the fragment D have a significant correlation with PLT count >280 × 10^9^/L suggesting a possible role between the increase in content of cleaved FGB in T tissues and PLT count at plasma level. Platelet activation and aggregation, in addition to accelerating coagulation, provide a bolus of secreted proteins, including fibrinogen, and granule contents to the immediate area, all of which help to initiate and accelerate the inflammatory response(s) by the host. It is interesting to note that soluble fibrin monomers have been proposed to enhance PLT adhesion to circulating tumor cells and, thus, facilitate metastatic spread [36,57]. In particular, activated PLTs, similarly to tumor cells, have receptors specific for binding with fibrinogen and fibrin fibers (GPIIb/IIIa). It cannot be excluded that these interactions may also occur in our tissues.

Results of the present study added new information regarding the association of blood parameters and hypercoagulability in GC with the FGB and its cleaved products, which we found differentially expressed in situ in our samples. The FGB forms we found may thus represent new candidate molecules for GC diagnosis, which could be further exploited in terms of possible interconnections between cancer and platelet/coagulation pathways. Recent proposals of many coagulation assays for prognostic value strengthen the importance of coagulation-related investigations in cancer clinics [42,43,58,59].

## 4. Materials and Methods

### 4.1. Patients and Tissues

A total of 28 tumor (T) and corresponding adjacent healthy normal (N) gastric tissue biopsies were collected at diagnosis from 28 patients enrolled at the CRO-IRCCS, National Cancer Institute of Aviano (PN), Italy CRO National Cancer institute (Table 1), following the approval by the Institutional Review Board (IRB) of CRO-IRCCS, National Cancer Institute of Aviano (PN), Italy (IRB number CRO-2014-03, 3 March 2014) and written informed consent of all the participating patients. These paired biopsies were stored at −80 °C until analyses. Clinical and laboratory evaluations of all patients are reported in Table 1 and Table 2, Table A1 and Table A2.

Patients were enrolled between April 2014 and December 2016 and stratified dependently on tumor depth according to the stomach TNM clinical classification [60], evaluated by ultrasound endoscopy.

### 4.2. Blood Sample Analysis

Venous blood samples in citrated tubes (0.109 M) were collected at diagnosis before the initiation of the treatments. Test performed were: prothrom-bin time–international normalized ratio (PT-INR), activated partial thromboplastin time (APTT), fibrinogen by Clauss clotting method (Siemens-Dade Behring Healthcare Diagnostics, Marburg, Germany). White blood cells (WBC) and platelet count were measured in EDTA blood samples with a ADVIA2120 Analyser (Siemens).

We considered the following blood biomarker data: white blood cells (WBC); absolute neutrophil count (ANC); absolute lymphocyte count (ALC); ANC/ALC ratio (N/R); platelet count (PLT); prothrombin time (PT-INR); fibrinogen (FIB); activated partial thromboplastin time (APTT).

For 6 patients these analyses were not performed because plasma was not available.

### 4.3. Sample Preparation and Grouping

Soluble proteins were extracted from the frozen biopsies as previously reported [61]. Briefly, frozen tissue samples were lysed in 200 µL cold lysis buffer (4% (*w*/*v*) CHAPS, 7 M Urea, 2 M Thiourea, 30 mM Tris, pH 8.5) with a protease inhibitor cocktail (Sigma-Aldrich, St. Louis, MO, USA), homogenated with a sample grinding kit (GE Healthcare, Uppsala, Sweden) and prepared for two-dimensional electrophoresis (2DE) with 2D Clean-Up Kit (GE Healthcare, Uppsala, Sweden). Protein concentrations were determined with the commercial Bradford reagent (Bio-Rad, Milan, Italy). 

Samples were grouped into two groups: “Group I” and “Group II”, including patients with primary tumors classified as either “T1 or T2”, or “T3 or T4”, respectively. Other comparison groupings were based on: (i) anatomical subsite (corpus or antrum); (ii) histological type (diffuse or intestinal); (iii) age (< or >50 years); and (iv) sex (male or female).

### 4.4. One-Dimensional Electrophoresis (1DE) and Immunoblotting Anti-Fibrinogen β Chain

The presence of FGB and its possible differential abundance were first investigated by immunoblotting analyses. Within each T group (I and II), proteins extracted from either GC or C tissues were pooled (3 patients per pool). Ten µg of proteins per pool were fractionated on 12% Criterion TGX Stain-Free gels and, after gel image acquisition upon fluorescence excitation with the Chemidoc system (Bio-Rad, Milan, Italy), electrotransferred onto nitrocellulose membranes. Membranes were incubated with the monoclonal antibody anti-FGB [1F9] (1:500; GeneTex, Irvine, CA, USA). Antibody-bound proteins were detected by enhanced chemiluminescence using the Chemidoc system after incubation with ECL HRP-conjugated secondary antibodies (1:10,000 dilution, Santa-Cruz, CA, USA) and reaction with ClarityTM Western ECL Substrate (Bio-Rad, Milan, Italy). Because of the lack of universal house-keeping genes to be used as sample loading control in GC [62], image of the stain-free gel acquired before its transfer was used as control for equal protein loading among samples.

### 4.5. Validation of the 1DE Bands Cross-Reacting with Fibrinogen β Chain

Pooled protein T extracts (10 µg per lane) were separated by 1DE, and images of blue-stained gel were acquired with the Chemidoc system. A total of 10 gel portions in the MW range between 75 and 37 kDa containing proteins cross-reacting with the FGB antibody (Figure 2, rectangle and numbered lanes) were excised, reduced by incubation with 10 mM dithiothreitol (1 h at 57 °C), and alkylated with 55 mM iodoacetamide (45 min at room temperature). Samples were further washed with NH_4_HCO_3_, dehydrated, trypsin digested and processed for LC-MS/MS analyses using a LTQ XL-Orbitrap ETD equipped with a HPLC NanoEasy-PROXEON (Thermo Fisher Scientific, Waltham, MA, USA). Database searches were done with the MASCOT search engine version 2.3 against SwissProt and NCBInr (Matrix Science, London, UK), and the presence of FGB was searched among first 15 report hits.

### 4.6. Two-Dimensional Difference in Gel Electrophoresis (2D-DIGE) and Immunoblotting Anti-Fibrinogen β Chain

The entire project consisted of 25 gels detailed in Table A1, each gel containing two protein extracts (25 µg per extract) from both T and N tissues of the same patient, respectively, each labeled with Cy3 or Cy5 with the internal standard (Cy2) representative of the all samples analyzed. In two gels, when protein tissue concentration was not sufficient (gels 5 and 10, Table A1), only proteins from one type of biopsy were analyzed per patient, and protein extracts from two different patients were comigrated. 

Cyanine dyes were used for protein labeling (CyDye DIGE Fluor minimal dyes; GE Healthcare, Uppsala, Sweden). Proteins were separated by isoelectrofocusing (IEF) on 3–10 pH gradient dry strips (IPG, GE Healthcare, Uppsala, Sweden) and then on 8–16% Criterion TGX precast midi protein gels (Bio-Rad, Milan, Italy). After 2DE and image gel acquisition (Amersham Typhoon; GE Healthcare, Uppsala, Sweden), differential analysis (DeCyder software version 6.5, GE Healthcare, Uppsala, Sweden) was performed.

One 2DE gel containing 50 µL of two protein extracts (N and T) labeled with cyanines was scanned, its image being loaded to the Decyder project, and immediately after electrotransferred onto a nitrocellulose membrane, which was then processed for revelation of FGB as described above. The 2DE spots visualized in the nitrocellulose membrane as cross-reacting with the anti-FGB antibody were then found in the corresponding Decyder gel, and further image differential gel analyses focused on these only spots of interest. This immunoblotting analysis was performed in triplicate. 

### 4.7. Database Searches for Fibrinogen β Chain Sequence and Post-Translational Modifications

To better interpret the experimental data about FGB isoforms, we focused FGB amino acid sequence and its possible enzymatic cleavage available in UniProtKB (http://www.uniprot.org/uniprot/P02675). Possible post-translational modifications were searched with the iPTMnet integrated resource ([63]; http://proteininformationresource.org/iPTMnet) and GeneCards^®^ human gene database (http://www.genecards.org), using FGB as substrate. The profiles of FGB-spots were compared with those either found after searches in the bibliographic database PubMed (https://www.ncbi.nlm.nih.gov/pubmed/) or available on-line on databases of 2D gel reference maps (DOSAC-COBS-2DPAGE, OGP, REPRODUCTION-2DPAGE, SWISS-2DPAGE, UCD-2DPAGE).

### 4.8. Image Analysis and Statistics

First, in intragroup comparisons within Groups I and II, we compared the abundances of a selected set of spots in N versus T tissues of the same patient (Table 3). While in intergroup comparisons, N tissue proteome profiles of Group I were compared with N ones of Group II (Table 3) and, similarly, T tissue proteome profiles of Group I were compared with T ones of Group II (Table 4). Secondly, we focused on the only T gastric tissues, and we compared the expressions of our spots of interest among patients depending on: (i) anatomical subsites (corpus versus antrum), (ii) histological types (intestinal versus diffuse) and (iii) age (> versus <50 years old) (Table 4). 

Gel image pairs were processed by the Decyder Differential In-gel Analysis (DIA) module to co-detect and differentially quantify the protein spots in the images; the internal standard sample was used as a reference to normalize the data, so the rest of the normalized spot maps could be compared among them. While the Biological variation analysis (BVA) module allowed to perform a gel-to-gel matching of spots across multiple gels, allowing quantitative comparison of protein expression. 

The BVA workspace was imported in the Decyder EDA (Extended Data Analysis) module to perform univariate statistical analyses of FGB spot expression. Paired t-test was performed to compare spot expression in normal versus tumor samples collected from the same patient. While Group I (N and T) to Group II (N and T) comparison of spot differential expression was analyzed using independent statistical tests based on average spot volume ratio with One-way Anova corrected with false discovery rate (default setting). Intergroup comparison depending on (i) gender, (ii) age, (iii) cancer histological type and (iv) location were performed using independent statistical tests with Student’s t-test, since these factors between Groups I and II were not statistically different. Based on average spot volume ratio, spots for which relative expression changed at least 1.5-fold (increase or decrease) at 95% confidence level (*p* < 0.05) were considered to be significant. Sample size was calculated considering an intergroup spot fold change of 1.5, a σ value of 50, an α value of 0.05, and a power of the test of 0.80. The sample size for each group resulted 8, and it was enlarged in the case of Group II where preliminary analyses showed a higher FGB content.

### 4.9. Correlation Analysis of Blood Parameters

Log standard abundance data of spots 402, 526, 548 and 501 in both T and N tissue proteomes of Group I and II patients were correlated with blood markers.

We focused on the only 5 patients of our cohort having the abundance of FGB spots in their T tissue proteome maps and PLT count >280 × 10^9^/L (asterisks in Table A2).

We used a PLT cut-off value of 280 × 10^9^/L estimating the sample mean (208 × 10^9^/L) and two standard deviations (±70 × 10^9^/L) in a cohort of healthy subjects.

## 5. Conclusions

By a targeted comparative proteomics, we succeeded in revealing an *in situ* differential expression of both the entire and the cleaved FGB forms in GC tissues, depending on cancer development, location, as well as pathological stage. Some of the found FGB forms were associated with blood parameters linked to hypercoagulability. The FGB-related proteins may thus represent new candidate(s) for GC diagnosis, which may be further exploited in terms of possible interconnections between cancer and coagulation pathways. 

## Figures and Tables

**Table 1:** Clinicopathological characteristics of the 28 patients affected by gastric cancer included in the study (n.s., statistically not significant for *p* > 0.05).

Characteristic	uT1–T2	uT3–T4	*p*-Value
Gender			n.s.
Male	4	9	
Female	5	10	
Age, years			n.s.
<50	3	4	
>50	6	15	
Histological type			n.s.
Intestinal	4	4	
Diffuse	4	13	
Other	1	2	
Location			n.s.
Corpus	4	14	
Antrum	4	4	
Cardias	1	1	

uT, endoscopic ultrasound evaluation of tumor depth: uT1, invasion of lamina propria, muscolaris mucosae or submucosa; uT2, invasion of muscolaris propria; uT3, invasion of subserosa; uT4, penetration of serosa or invasion of adjacent structures.

**Table 2:** Blood count parameters of gastric cancer (GC)-affected patients (WBC, white blood cells; ANC, absolute neutrophil count; ALC, absolute lymphocyte count; N/R, ANC/ALC ratio; PLT, platelet count; PT-INR, prothrombin time–international normalized ratio; FIB, fibrinogen level; APTT, activated partial thromboplastin time). Values at diagnosis refer to 22 patients.

Parameter	Mean (±SD)
WBC (×10^9^/L)	6.33 ± 1.96
ANC(×10^9^/L)	3.93 ± 1.54
ALC (×10^9^/L)	1.83 ± 0.74
N/R	2.54 ± 1.71
PLT (×10^9^/L)	258 ± 114.90
PT-INR	1.00 ± 0.05
FIB g/L	2.97 ± 46
APTTs	29 ± 3

**Table 3:** Comparison of 13 fibrinogen β chain (FGB) spot abundance between tumor-affected (T) and not-affected (N) gastric tissues of patients grouped into “Group I” or “Group II” according to their uT stage (“Group I”: T1 and T2; “Group II”: T3 and T4).

Comparison Groups		Spots												
		383	397	398	400	393	396	402	403	404	405	501	526	548
•	N “Group II” versus N “Group I”	1	**−1.6**	−1.3	−1.0	1.1	1.1	1.5	1.2	−1.0	1.1	1.1	1.0	1.4
•	T “Group I” versus N “Group I”	−1.3	**−1.5**	**−1.5**	−1.1	**1.5**	**1.5**	**2.0**	1.3	**1.8**	**1.6**	1.6	**1.5**	**1.6**
•	T “Group II” versus N “Group II”	−1.3	−1.2	−1.2	−1.3	**1.5**	1.2	**3.2**	**1.1**	**2.8**	**1.8**	1.22	**1.82**	**1.75**

Spot variation in abundance is expressed as average spot volume ratio; values >1 refer to an increase, while values <−1 refer to a decrease. Statistically significant average spot volume ratios <1.5 or >1.5 (One-way Anova; *p* < 0.05) are indicated in bold.

**Table 4:** Intergroup comparison of FGB spots abundance in affected tumor-samples.

Groups		Spots												
		383	397	398	400	393	396	402	403	404	405	501	526	548
•	“Tumor staging” Group II (nr = 19) versus Group I (nr = 9)	−1.3	1.0	−1.2	−1.3	1.4	1.2	**3.2**	1.1	**2.8**	1.8	1.2	**1.8**	**1.8**
•	“Anatomical subsites” Antrum (nr = 8) versus. corpus (nr = 18)	−1.0	**−1.9**	**−2.0**	1.4	**1.7**	**1.7**	**3.4**	**1.9**	**2.8**	**2.3**	1.9	−1.5	−1.7
•	“Histological subtypes” Diffuse (nr = 18) versus intestinal (nr = 7)	−1.1	1.0	−1.3	−1.3	−1.3	−1.1	−2.1	1.0	−2	−1.7	**3.0**	1.3	−1.1
•	“Age” >50 years (nr = 19) versus <50 years (nr = 7)	1.1	1.4	1.2	1.1	1.1	−1.1	**3.6**	−1.0	1.8	2.0	−1.1	1.1	1.2
•	“Sex” Female (nr = 15) versus male (nr = 13)	1.3	2.0	1.8	1.3	−1.0	1.0	−1.0	−1.1	1.2	1.1	**−2**	−1.3	1.0

Spot variation in abundance is expressed as average spot volume ratio. Statistically significant spot volume ratio <1.5 or >1.5 (Student’s *t*-test; *p <* 0.05) were indicated in bold. Values >1.5 refer to an increase, while values <−1.5 refer to a decrease in the average spot volume ratio.

## Abbreviations

1DE	one-dimensional electrophoresis
2DE	two-dimensional electrophoresis
ALC	absolute lymphocyte count
ANC	absolute neutrophil count
APTT	activated partial thromboplastin time
APTT RATIO	ratio of APPT
BVA	biological variation analysis
DIA	decyder differential in-gel analysis
DIGE	difference gel electrophoresis
FGB	fibrinogen β chain
FIB	fibrinogen level
GC	gastric cancer
IEF	isoelectrofocusing
N/R	ANC/ALC ratio
PLT	platelet count
PT	prothrombin time
WBC	white blood cells

## Appendix A

**Table A1:** Clinical details of the gastric cancer (GC)-affected patients and corresponding protein pairs extracted from both normal (N) and gastric cancer-affected (T) tissues, labelled with either Cy3 or Cy5 dyes and mixed with the Cy2-labelled internal standard for two-dimensional difference in gel electrophoresis, as described in ‘Material and methods’ section.

Patient nr.	Age	Male/Female ^a^	uT	uN ^b^	Histological Classification ^b^	Anatomical Subsites	Tissue ^c^	Cye Dye	Gel nr.
1	54	M	3	N+	diffuse	corpus	N	cy3	1
							T	cy5	
2	50	F	3	N+	diffuse	antrum	N	cy3	2
							T	cy5	
3	44	F	3	N+	diffuse	corpus	N	cy3	3
							T	cy5	
4	39	F	3	N+	diffuse	corpus	N	cy3	4
							T	cy5	
5	68	M	3	N0	diffuse	corpus	N	cy3	5
6	52	M	3	N+	n.a.	corpus	T	cy5	
7	55	M	3	N+	diffuse	corpus	N	cy5	6
							T	cy3	
8	81	F	3	N+	intestinal	antrum	N	cy5	7
							T	cy3	
9	74	M	3	N+	diffuse	antrum	N	cy5	8
							T	cy3	
10	65	M	4	N+	diffuse	corpus	N	cy5	9
							T	cy3	
11	71	F	3	N0	diffuse	antrum	N	cy5	10
12	69	M	3	N+	intestinal	corpus	T	cy3	
13	60	F	4	n.a.	other	corpus	N	cy5	11
							T	cy3	
14	68	F	4	N0	intestinal	corpus	N	cy5	12
							T	cy3	
15	54	M	3	N+	diffuse	cardia	N	cy5	13
							T	cy3	
16	70	F	2	n.a.	intestinal	antrum	N	cy5	14
							T	cy3	
17	76	F	1	N0	intestinal	antrum	N	cy3	15
							T	cy5	
18	59	M	1	N0	other	cardia	N	cy3	16
							T	cy5	
19	66	F	1	N0	diffuse	corpus	N	cy5	17
							T	cy3	
20	48	M	2	N0	intestinal	corpus	N	cy5	18
							T	cy3	
21	36	F	1	N0	intestinal	corpus	N	cy5	19
							T	cy3	
22	59	M	1	N+	diffuse	corpus	N	cy5	19
							T	cy3	
23	69	F	3	N0	n.a.	corpus	N	cy3	20
							T	cy5	
24	58	M	3	N+	diffuse	corpus	N	cy3	21
							T	cy5	
25	45	F	3	N+	diffuse	corpus	N	cy5	22
							T	cy3	
26	68	M	1	N0	diffuse	antrum	N	cy5	23
							T	cy3	
27	50	F	1	N0	diffuse	antrum	N	cy5	24
							T	cy3	
28	55	F	3	N+	diffuse	corpus	N	cy5	25
							T	cy3	

^a^ M, male, F, female; ^b^ n.a., not available; ^c^ N, normal tissue; T, GC-affected tissue.

**Table A2:** Blood biomarkers of gastric cancer-affected patients (WBC, white blood cells; ANC, absolute neutrophil count; ALC, absolute lymphocyte count; N/R, ANC/ALC ratio; PLT, platelet count; PT, prothrombin time; FIB, fibrinogen level; APTT activated partial thromboplastin time; APTT RATIO, the ratio of APPT). Values at diagnosis refer to 22 patients.

Patient nr. ^a^	WBC × 10^9^/L	PLT × 10^9^/L	ANC × 10^9^/L	ALC × 10^9^/L	N/R	PT-INR	FIB g/L ^b^	APTTs	APTT RATIO
1	4.65	221	3	1.3	2.31	0.93	2.91	27	0.83
2	1.19	57	0.7	0.4	1.75	0.93	2.76	26	0.86
3 *	5.23	379	4.5	0.7	6.43	1.13	2.76	26	0.88
4	7.9	272	4.2	2.6	1.62	1	3.54	33	1.1
5	7.61	174	4.1	2.9	1.41	0.99	2.20	28	0.93
6 *	8.95	624	5.3	2.7	1.96	0.95	n.a.	27	0.89
7	6.29	182	3.4	2	1.70	0.99	2.95	34	1.14
8	4.74	184	2.6	1.8	1.44	1	2.76	31	1.03
9 *	7.19	283	4.3	2	2.15	0.96	3.34	29	0.95
10	9.61	137	7.8	1.1	7.09	1.03	3.01	24	0.79
11	7.92	330	5.3	2	2.65	0.99	n.a.	32	1.04
12 *	7.57	421	3.9	3.1	1.26	0.95	2.96	31	1.03
13	8.12	231	6.1	1.2	5.08	1.02	3.37	25	0.83
14	5.11	245	2.7	1.9	1.42	0.92	3.57	24	0.79
15	6.74	212	5.1	1.1	4.64	1.04	3.68	32	1.06
16	4.8	234	2.4	2	1.20	1.06	3.03	29	0.95
17	4,1	215	2.4	1.3	1.85	1.03	3.45	30	1.01
18 *	7.72	283	4.1	2.8	1.46	1.03	3.37	32	1.07
19	7.63	233	5.5	1.6	3.44	0.97	2.16	27	0.89
20 *	6.71	345	3.3	2.7	1.22	1.02	2.56	29	0.97
21	5.05	235	3.2	1.6	2.00	0.95	2.56	27	0.91
22	4.51	177	2.6	1.5	1.73	1.01	2.26	28	0.95

^a^ asterisk indicates patients with PLT count upper the cut-off value: 280 × 10^9^/L; ^b^ n.a., not available.

**Table A3:** Details of fibrinogen β chain identification by mass spectrometry and database searches in protein extracts of gastric cancer tissues separated by one-dimensional electrophoresis. The MASCOT scores are reported for the only gel portions resulting to contain fibrinogen β chain.

Gel Portion nr.^a^	Database	Accession	Description	Score/Seq. Coverage %	Start-End	Peptide	Matches/Sequences
4	NCBInr	gi|119625336	Fibrinogen β chain, isoform CRA_b	239/29	42–48 50–69 161–175 197–203 220–226 228–244	R.GHRPLDK.K K.REEAPSLRPAPPPISGGGYR.A K.DNENVVNEYSSELEK.H R.SILENLR.S K.ECEEIIR.K K.GGETSEMYLIQPDSSVK.D	11/7
5	SwissProt	FIBB_HUMAN	Fibrinogen β chain	376/28	45–51 53–72 164–178 200–206 212–224 240–246 248–267 301–313 354–367 368–374 411–421 484–491	R.GHRPLDK.K K.REEAPSLRPAPPPISGGGYR.A K.DNENVVNEYSSELEK.H R.SILENLR.S K.LESDVSAQMEYCR.T K.ECEEIIR.K K.GGETSEMYLIQPDSSVKPYR.V K.QGFGNVATNTDGK.N K.AHYGGFTVQNEANK.Y K.YQISVNK.Y R.DNDGWLTSDPR.K K.IRPFFPQQ.-	19/12
7 *	SwissProt	FIBB_HUMAN	Fibrinogen β chain	269/15	164–178 212–224 225–239 301–313 354–367 484–491	K.DNENVVNEYSSELEK.H K.LESDVSAQMEYCR.T R.TPCTVSCNIPVVSGK.E K.QGFGNVATNTDGK.N K.AHYGGFTVQNEANK.Y K.IRPFFPQQ.-	6/5
8 *	SwissProt	FIBB_HUMAN	Fibrinogen β chain	340/19	164–178 212–224 225–239 240–246 248–267 301–313 354–367	K.DNENVVNEYSSELEK.H K.LESDVSAQMEYCR.T R.TPCTVSCNIPVVSGK.E K.ECEEIIR.K K.GGETSEMYLIQPDSSVKPYR.V K.QGFGNVATNTDGK.N K.AHYGGFTVQNEANK.Y	8/7
9 *	SwissProt	FIBB_HUMAN	Fibrinogen β chain	477/21	164–178 212–224 225–239 240–246 248–267 301–313 354–367 484–491	K.DNENVVNEYSSELEK.H K.LESDVSAQMEYCR.T R.TPCTVSCNIPVVSGK.E K.ECEEIIR.K K.GGETSEMYLIQPDSSVKPYR.V K.QGFGNVATNTDGK.N K.AHYGGFTVQNEANK.Y K.IRPFFPQQ.-	13/11

^a^ gel portion nr., gel portion numbers refer to Figure 1c; *, gel portions containing the protein sequence 164–491, the fragment D of FGB, coming from plasmin cleavage in 163–164.
